# Supplementary material for: A novel mechanism for dissimilatory nitrate reduction to ammonium in Acididesulfobacillus acetoxydans
Source: mSystems. 2024 Feb 7;9(3):e00967-23. doi: 10.1128/msystems.00967-23 (PMC10949509; doi:10.1128/msystems.00967-23)
Supplement: File S1 — Growth of Acididesulfobacillus acetoxydans with nitrate and glycerol. [file msystems.00967-23-s0001.docx]

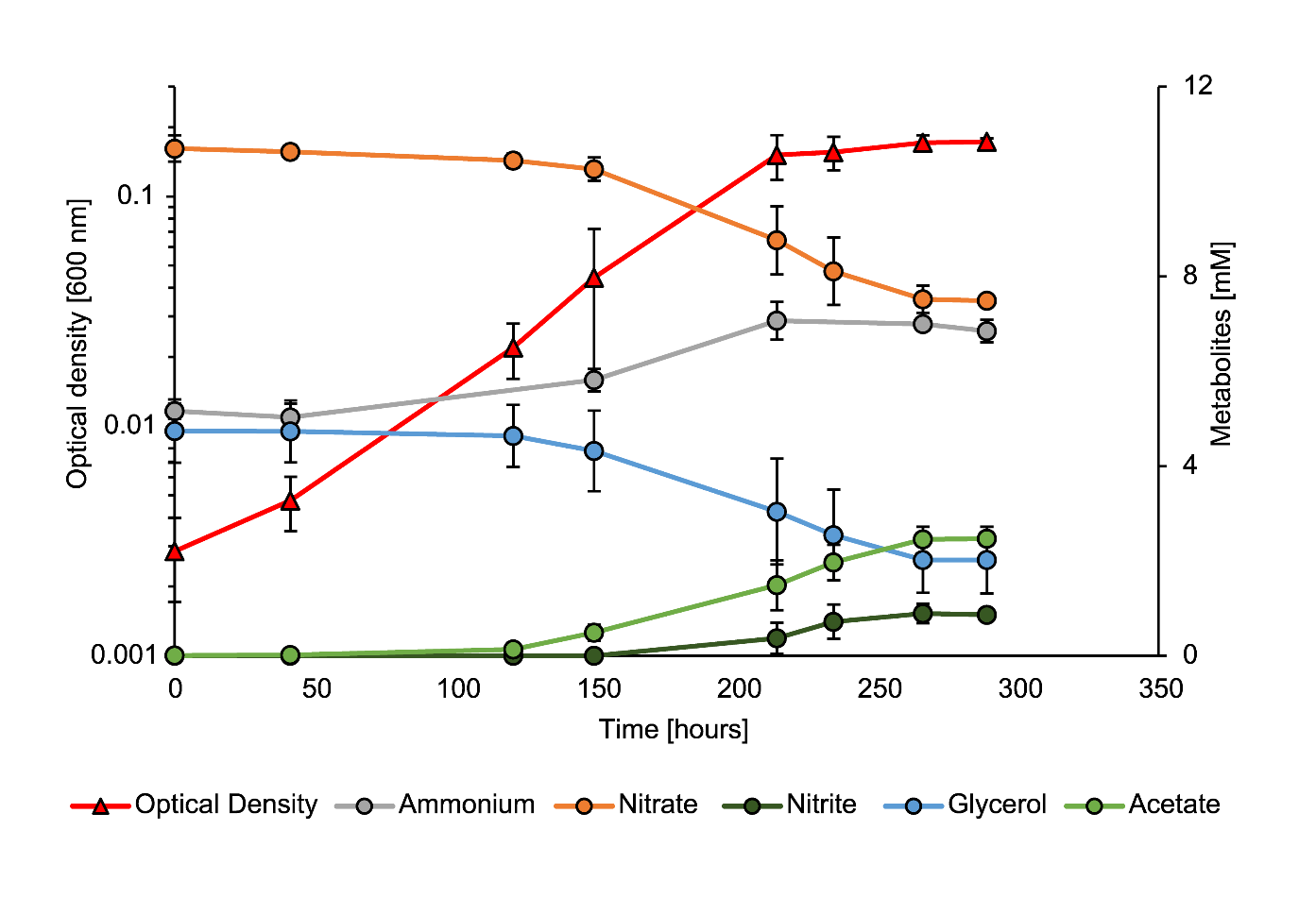
**Supplementary File S1.** Growth of *Acididesulfobacillus acetoxydans* with nitrate and glycerol

**Supplementary Figure S1. Growth of A. acetoxydans (quadruplicate) reducing NO_3_^-^ to NO_2_^-^ and NH_4_**^+^. Error bars indicate standard deviation. Growth halted after accumulation of approximately 0.8 mM NO_2_^-^.

**Supplementary Table S1.** Supplementary Figure S1.1 depicted in table format with nitrogen balances and glycerol to acetate ratios of A. acetoxydans growing on nitrate

| **Time [h]** | **NO_3_^-^ [mM]** | **NO_2_^-^ [mM]** | **NH_4_^+^ [mM]** | **N-balance** | **Glycerol [mM]** | **Acetate [mM]** | **Glycerol : Acetate** |
| --- | --- | --- | --- | --- | --- | --- | --- |
| 0 | 10.7 | 0.0 | 5.2 | 100% | 4.74 | 0.01 | 100% |
| 41 | 10.6 | 0.0 | 5.0 | 99% | 4.73 | 0.02 | 100% |
| 149 | 10.3 | 0.0 | 5.8 | 101% | 4.32 | 0.49 | 101% |
| 214 | 8.8 | 0.4 | 7.1 | 102% | 3.04 | 1.49 | 95% |
| 266 | 7.5 | 0.9 | 7.0 | 97% | 2.02 | 2.46 | 94% |
| 289 | 7.5 | 0.9 | 6.8 | 96% | 2.02 | 2.47 | 95% |
