# Supplementary material for: A novel mechanism for dissimilatory nitrate reduction to ammonium in Acididesulfobacillus acetoxydans
Source: mSystems. 2024 Feb 7;9(3):e00967-23. doi: 10.1128/msystems.00967-23 (PMC10949509; doi:10.1128/msystems.00967-23)
Supplement: File S2 — Resting cell experiments of Acididesulfobacillus acetoxydans. [file msystems.00967-23-s0002.docx]

**Supplementary File S2.** Resting cell experiments of *A. acetoxydans* with various electron acceptors and 2 mM of electron donor.


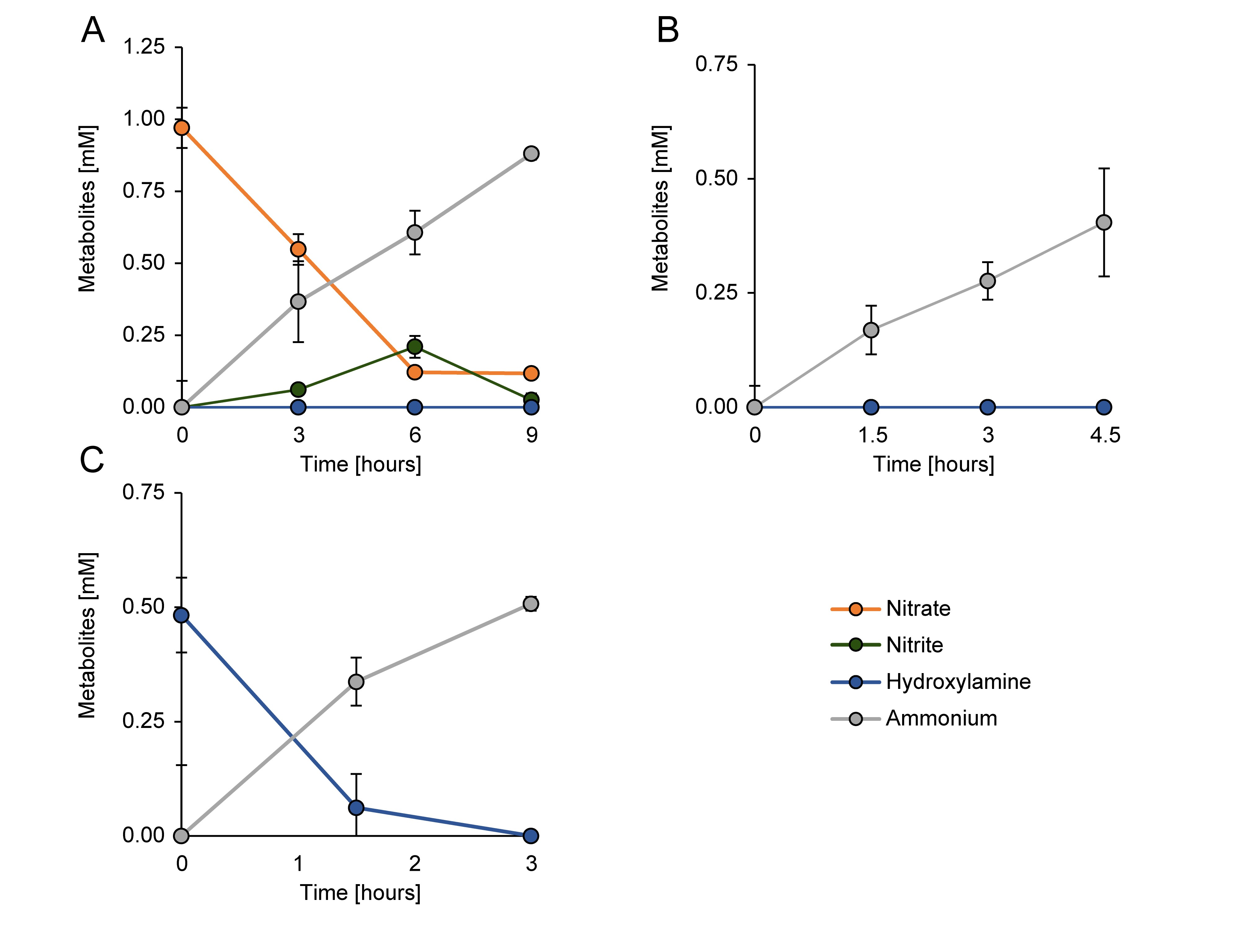


**Figure S2. Resting cell experiments of A. acetoxydans with various electron acceptors and 2 mM of electron donor.** A: Nitrate reduction (1 mM) to ammonium accumulates the intermediate nitrite which is later further reduced. B: Nitrite reduction (0.5 mM) to ammonium. C: Hydroxylamine (0.5 mM) is completely reduced to ammonium. Error bars represent the standard deviation. For clarity, ammonium is depicted as the surplus on top of the already present ammonium at the start of each resting cell experiment.
