# Supplementary material for: A novel mechanism for dissimilatory nitrate reduction to ammonium in Acididesulfobacillus acetoxydans
Source: mSystems. 2024 Feb 7;9(3):e00967-23. doi: 10.1128/msystems.00967-23 (PMC10949509; doi:10.1128/msystems.00967-23)
Supplement: File S4 — Upregulated molybdopterin containing oxidoreductases. [file msystems.00967-23-s0004.docx]

**Supplementary File S4.** Upregulated molybdopterin containing oxidoreductases

Two gene clusters, DEACI_2560-2564 and DEACI_3268-3271, were upregulated in the transcriptome when nitrate was used as final electron acceptor (Supplementary Table S4).

**Table S4.** Transcriptome and proteome data for the two upregulated clusters of molybdopterin containing oxidoreductases of A. acetoxydans under nitrate and sulfate reducing conditions.

| **Locus tag** | **Annotation** | **Transcriptome** | | **Proteome** | |
| --- | --- | --- | --- | --- | --- |
|  |  | **TPM N** | **TPM S** | **LFQ N** | **LFQ S** |
| DEACI_2560 | sulfite reductase (NADPH) | 1510 | 145 | 8.9 | 7.1 |
| DEACI_2561 | RmlC-like jelly roll fold | 934 | 172 | - | - |
| DEACI_2562 | nitrate reductase | 185 | 23 | 9.0 | 7.3 |
| DEACI_2563 | 4Fe-4S ferredoxin-type, iron-sulphur binding domain protein | 149 | 5 | 8.8 | 7.2 |
| DEACI_2564 | Polysulphide reductase, NrfD | 159 | 10 | - | - |
| DEACI_3264 | RmlC-like jelly roll fold | 57 | 100 | 8.0 | 7.2 |
| DEACI_3268 | Nitrate reductase delta subunit | 352 | 30 | 8.0 | 7.1 |
| DEACI_3269 | Polysulphide reductase, NrfD | 304 | 24 | 7.7 | 7.1 |
| DEACI_3270 | 4Fe-4S ferredoxin-type, iron-sulphur binding domain protein | 325 | 26 | 8.9 | 8.0 |
| DEACI_3271 | Prokaryotic molybdopterin oxidoreductases 4Fe-4S domain protein | 155 | 19 | 9.8 | 7.9 |

These complexes are characterized by an NrfD-like subunit, an iron-sulfur subunit, and a catalytic subunit. The NrfD-like subunits contain 8-10 transmembrane helices to anchor in the membrane; DEACI_2564 and DEACI_3269 are predicted to contain eight and ten transmembrane helices, respectively. The iron sulfur subunits are encoded by DEACI_2563 and DEACI_3270 with multiple [4Fe4S] domains. The catalytic subunit is encoded by DEACI_2562, an 889 amino acid protein, containing a [4Fe4S] domain, a molybdopterin dinucleotide-binding domain and a molybdopterin oxidoreductase domain. The catalytic subunit of the other cluster, DEACI_3271, is an 826 amino acid protein which contains a small twin-arginine translocation sequence, a molybdopterin dinucleotide-binding domain and a molybdopterin oxidoreductase domain. DEACI_3268 encodes a TorD-like chaperone protein for maturation of DMSO reductase and facilitates insertion of the molybdenum cofactor. Near both gene clusters a Crp/Fnr family type regulator is encoded by DEACI_2561 and DEACI_3264. DEACI_2561 was upregulated in the transcriptome, but not detected in the proteome whereas DEACI_3264 was expressed in both conditions without changing expression levels in the transcriptome, but significantly increased in abundance during nitrate reduction in the proteome.
