# Supplementary material for: A novel mechanism for dissimilatory nitrate reduction to ammonium in Acididesulfobacillus acetoxydans
Source: mSystems. 2024 Feb 7;9(3):e00967-23. doi: 10.1128/msystems.00967-23 (PMC10949509; doi:10.1128/msystems.00967-23)
Supplement: File S5 — Multiple sequence alignment of NirA and DEACI_1836. [file msystems.00967-23-s0005.docx]

**Supplementary File S5.** Amino Acid multiple sequence alignment performed with ClustalW comparing *A. acetoxydans* DEACI_1836 to NirA of *Spinacia oleracea* (1), of *Phormidium laminosum* (2), *Mycobacterium tuberculosis* (3), *Synechococcus elongatus* (4) and *Pseudomonas aeruginosa* (5). The clustal consensus shows conserved residues and the conserved cysteine residues highlighted in gray coordinate via covalent bonds the four iron ions of the [4Fe4S] cluster and the iron of the siroheme. The conserved invariant residue asparagine highlighted in green is present in the active site and potentially involved in the electron transfer from ferredoxin to the [4Fe4S] cluster (3).

**10 20 30 40 50 60**

**....|....| ....|....| ....|....| ....|....| ....|....| ....|....|**

**S_olera-NirA**  MASLPVNKII PSSTTLLSSS NNNRRRNNSS IRCQKAVSPA AETAAVSPSV DAARLEPRVE 60

**P_lamin-NirA**  ---------- ---------- ---------- ---------M TSTVPAE--- ---------- 8

**M_tuber-NirA**  ---------- ---------- ---------- ---------M TTARPAKARN EGQWALG--- 18

**S_elong-NirA**  ---------- ---------- ---------- ---------M AQATATT--- ---------- 8

**P_aerug-NirA**  ---------- ---------- ---------- ---------M YQYDEYD--- ---------- 8

**DEACI_1836**  ---------- ---------- ---------- ---------- ---------- ---------- 1

**Clustal consensus** 1

**70 80 90 100 110 120**

**....|....| ....|....| ....|....| ....|....| ....|....| ....|....|**

**S_olera-NirA**  ERDGFWVLKE EFRSGINPAE KVKIEKDPMK LFIEDGIS-D LATLSMEEVD KSKHNKDDID 119

**P_lamin-NirA**  ---------- ---TSLNKFE KLKSEKD--G LAVKSELE-D FARLGWEAMD ET-----DRD 47

**M_tuber-NirA**  ---------- -HREPLNANE ELKKAGN--P LDVRERIENI YAKQGFDSID KT-----DLR 60

**S_elong-NirA**  ---------- ---EKLNKFE KLKLEKD--G LAVRDQIQ-H FASIGWEAMD PG-----DRE 47

**P_aerug-NirA**  ---------- ----QALVSE RVAQFRD--- -QIARRLDGE LSEEEFLPLR LQN------- 43

**DEACI_1836**  ---------- ---------- ---------- ---------- ---------- ---------- 1

**Clustal consensus** 1

**130 140 150 160 170 180**

**....|....| ....|....| ....|....| ....|....| ....|....| ....|....|**

**S_olera-NirA**  VRLKWLGLFH RR-------- -------KHH YGRFMMRLKL PNGVTTSEQT RYLASVIKKY 164

**P_lamin-NirA**  HRLRWMGVFF RP-------- -------VSQ -GKFMLRMRI PNGILTSGQI RVLAEVVERY 91

**M_tuber-NirA**  GRFRWWGLYT QREQGYDGTW TGDDNIDKLE AKYFMMRVRC DGGALSAAAL RTLGQISTEF 120

**S_elong-NirA**  HRLKWLGIFW RP-------- -------VTP -GRFMARLRI PSGILQSQQL NALANFLQRY 91

**P_aerug-NirA**  ------GLYL QK-------- ---------- -HAYMLRVAI PYGTLSAPQL RALAHVARHY 78

**DEACI_1836**  ---------- ---------- ---------- -MRFTLDMTL QDIMAAN--- ---------- 16

**Clustal Consensus** : : 2

**190 200 210 220 230 240**

**....|....| ....|....| ....|....| ....|....| ....|....| ....|....|**

**S_olera-NirA**  GKDGCADVTT RQNWQIRGVV LPDVPEIIKG LESVGLTSLQ SGMDNVRNPV GNPLAGIDPH 224

**P_lamin-NirA**  GEDGNADITT RPNLQLRGIR LEDIPDIFRR FEQAGLTSIQ SGMDNVRNIT GSPVAGIDAD 151

**M_tuber-NirA**  ARD-TADISD RQNVQYHWIE VENVPEIWRR LDDVGLQTTE ACGDCPRVVL GSPLAGESLD 179

**S_elong-NirA**  GDQASIDITT RQNLQLRGLL LEDTPEFLER LHAVGLTSVQ SGMDNVRNIT GSPVAGLDAA 151

**P_aerug-NirA**  DRG-YGHFTT RQNIQFNWIE LEQVGDILEH LAGAQMHAIQ TSGNCVRNIT TEAFAGVAAD 137

**DEACI_1836**  ---------- ---------- ----PKTVDA MQEMGLHCLG CPFSIKENLG N--------- 43

**Clustal consensus** . : : . . 4

**250 260 270 280 290 300**

**....|....| ....|....| ....|....| ....|....| ....|....| ....|....|**

**S_olera-NirA**  EIVDTRPFTN LISQFVTANS RGNLSITNLP RKWNPCVIGS HDLYEHPHIN DLAYMPATK- 283

**P_lamin-NirA**  ELIDTRGLVR KVQDMITNNG EGNPSFSNLP RKFNIAIAGC RDNSVHAEIN DIAFVPAYK- 210

**M_tuber-NirA**  EVLDP----T WAIEEIVRRY IGKPDFADLP RKYKTAISGL QD--VAHEIN DVAFIGVNH- 232

**S_elong-NirA**  ELFDTRSLIQ ALQDDLTAAG QGNSEFTNLP RKFNIAIEGG RDNSIHAEIN DLAFTPAYQ- 210

**P_aerug-NirA**  EWTDP----R PLAEILRQWS TVNPEFLFLP RKFKIALSSA VEDRAAVQMH DIGLYLYRHP 193

**DEACI_1836**  ---------- ---------- ---------- ---AAQMHGI DPQALLARVN EVE------- 63

**Clustal Consensus** : . .:: :: 9

**310 320 330 340 350 360**

**....|....| ....|....| ....|....| ....|....| ....|....| ....|....|**

**S_olera-NirA**  NGK-FGFNLL VGGFFSIKRC EEAIPLDAWV SA-EDVVPVC KAMLEAFRDL GFRGNRQKCR 341

**P_lamin-NirA**  DGK-LGFNVL VGGFFSAKRC EAAVPLNAWV DP-RDVVALC EAILIVYRIT GCGANRQKSR 268

**M_tuber-NirA**  PEHGPGLDLW VGGGLSTN-P MLAQRVGAWV PL-GEVPEVW AAVTSVFRDY GYRRLRAKAR 290

**S_elong-NirA**  DGT-LGFNVW VGGFFSSTRV APAIPLNAWV PADHSVIRLS RAILEVFRDN GSRGNRQKTR 269

**P_aerug-NirA**  DAGELRLRVL VGGGLGRTPM LGQVIRDDLP WQ--HLLSYV EAILRVYNRY GRRDNKYKAR 251

**DEACI_1836**  ---------- ---------- ---------- ---------- ---------Q GEMSAQAKAN 74

**Clustal consensus** * : * . 12

**370 380 390 400 410 420**

**....|....| ....|....| ....|....| ....|....| ....|....| ....|....|**

**S_olera-NirA**  MMWLIDELGM EAFRGEVEKR MPEQ------ ---------- ---------V LERASSEE-- 374

**P_lamin-NirA**  LMWLIDEWGM DKFRAEVEQQ LGHP------ ---------- ---------L QTAAPK---- 299

**M_tuber-NirA**  LKFLIKDWGI AKFREVLETE YLKR------ ---------- ---------P LIDGPAP--- 322

**S_elong-NirA**  LMWLIDEWGI ERFRQVVSEA YGAP------ ---------- ---------L AAAAP----- 299

**P_aerug-NirA**  IKILVKALGI EAFAREVEEE WQHLRDGPAQ LTAEECQRVA ERFVLPRYLP PADGELAYGS 311

**DEACI_1836**  SQPEASVLQM DKKTYAIAPH IPAG------ ---------- ---------- ---------- 98

**Clustal consensus** . : : 14

**430 440 450 460 470 480**

**....|....| ....|....| ....|....| ....|....| ....|....| ....|....|**

**S_olera-NirA**  --LVQKDWER REYLGVHPQK QQGLSFVGLH IPVGR----- -LQADEMEEL ARIADVYGSG 426

**P_lamin-NirA**  ---DEILWDK RDHIGIHAQK KPGLNYVGLL VPVGR----- -LYAP-MFDL ARIAEVYGDG 349

**M_tuber-NirA**  ----EPVKHP IDHVGVQRLK N-GLNAVGVA PIAGR----- -VSGTILTAV ADLMARAGSD 371

**S_elong-NirA**  ---ELMDWEK RDFLGVHPQK QAGLNFVGLH VPVGR----- -LTTEDLYEL ARLADTYGQG 350

**P_aerug-NirA**  ARAADPAFAR WASRNVQAHK VPGYASVVLS TKPGASAPPG DVTAEQMERV ADWAERYGFG 371

**DEACI_1836**  ---------- ---------- ---------- ---------- VATPAILRKI ADVAEKYQAA 118

**Clustal consensus** : : * 17

**490 500 510 520 530 540**

**....|....| ....|....| ....|....| ....|....| ....|....| ....|....|**

**S_olera-NirA**  ELRLTVEQN- IIIPNVENSK IDSLLNEPLL KERYSPEPPI LMKGLVACTG SQFCGQAIIE 485

**P_lamin-NirA**  EMRLTVERKR DHSRTCPMSS VASLLKEPLL -EKFSVSPGL LVRSLVSCTG AQFCNFALIE 408

**M_tuber-NirA**  RIRFTPYQK- LVILDIPDAL LDDLIAGLDA LGLQS-RPSH WRRNLMACSG IEFCKLSFAE 429

**S_elong-NirA**  EVRLTVEQN- VILTHIPDAQ LPTLLAEPLL -TRFSPQPAP LSRGTVSCTG SQYCNFALIE 408

**P_aerug-NirA**  EIRVAHEQN- LVLPDVRLEN LHALWREACA AGLGTPNQGL LS-DIIACPG GDYCALANAK 429

**DEACI_1836**  ALKVTTAQR- IAIVGLKPED VPKAWADLGM DPGHA--AGV CVRSVKVCPG NTFCKRGLQE 175

**Clustal consensus** ::.: :. : : . *.* :* . : 28

**550 560 570 580 590 600**

**....|....| ....|....| ....|....| ....|....| ....|....| ....|....|**

**S_olera-NirA**  TKARALKVTE EVQRLVSVTR -----PVRMH WTGCPNSCGQ VQVADIGFMG CMTRDENGKP 540

**P_lamin-NirA**  TKNRAMALIR ELESELELAR -----PVRIH WTGCPNSCGQ PQVADIGLMG TKVR-KDGKA 462

**M_tuber-NirA**  TRVRAQHLVP ELERRLEDIN SQLDVPITVN INGCPNSCAR IQIADIGFKG QMIDDGHGGS 489

**S_elong-NirA**  TKQRAIAIAQ SLEAELDLPR -----PVRIH WTGCPNSCGQ PQVADIGLMG AKVR-KDGQM 462

**P_aerug-NirA**  SIPIAQGIQQ RFEDLDHLHD IG---ELSLN ISGCMNACGH HHIGNIGILG VDKS-----G 481

**DEACI_1836**  TLTFGVELDK RYHGMPLPSK ------FKIA VAGCPNKCTD SATVDLGLMG ---------T 220

**Clustal consensus** : . : . . : ** * * ::*: * 40

**610 620 630 640 650 660**

**....|....| ....|....| ....|....| ....|....| ....|....| ....|....|**

**S_olera-NirA**  CEGADVFVGG RIGSDSHLG- DIYKKAVPCK DLVPVVAEIL INQFGAVPRE REEAE----- 594

**P_lamin-NirA**  TEGVDLYMGG KVGKHAELG- TCVQKGIPCD DLKPILRNLL IEHFGARPK- ---------- 510

**M_tuber-NirA**  VEGFQVHLGG HLGLDAGFGR KLRQHKVTSD ELGDYIDRVV RNFVKHRSEG ERFAQWVIRA 549

**S_elong-NirA**  VEGVDIFLGG KVGYDAHLG- EKAMTGVACE DLPDVLRQLL IERFGAQARS H--------- 512

**P_aerug-NirA**  SEWYQVTLGG AQGKDSALG- KVIGPSFSAA EVPAVIERIV ETFTDLRVGP ERFIDTFNRV 540

**DEACI_1836**  SKGYHLYVGG NGGVKPRLA- DLLWENLQES ERFPAVEAVI EYYKEKAKPQ ERLGRMLDRL 279

**Clustal consensus** : .: :** * .. :. . : : :: 51

**670 680**

**....|....| ....|....| .**

**S_olera-NirA**  ---------- ---------- - 594

**P_lamin-NirA**  ---------- ---------- - 510

**M_tuber-NirA**  EEDDLR---- ---------- - 555

**S_elong-NirA**  ---------- ---------- - 512

**P_aerug-NirA**  GLEPFKARVY ARMEEPA--- - 557

**DEACI_1836**  GLDELRGRVG KTLETLSSVK Q 300

**Clustal consensus** 51

**Supplementary File S5 references:**

1. Swamy U, Wang M, Tripathy JN, Kim SK, Hirasawa M, Knaff DB, Allen JP. 2005. Structure of spinach nitrite reductase: Implications for multi-electron reactions by the iron-sulfur:siroheme cofactor. Biochemistry 44:16054–16063.

2. Merchán F, Prieto R, Kindle KL, Llama MJ, Serra JL, Fernández E. 1995. Isolation, sequence and expression in *Escherichia coli* of the nitrite reductase gene from the filamentous, thermophilic cyanobacterium *Phormidium laminosum*. Plant Mol Biol 27:1037–1042.

3. Schnell R, Sandalova T, Hellman U, Lindqvist Y, Schneider G. 2005. Siroheme- and [Fe4-S4]-dependent NirA from *Mycobacterium tuberculosis* is a sulfite reductase with a covalent Cys-Tyr bond in the active site. J Biol Chem 280:27319–27328.

4. Luque I, Flores E, Herrero A. 1993. Nitrite reductase gene from *Synechococcus* sp. PCC 7942: homology between cyanobacterial and higher-plant nitrite reductases. Plant Mol Biol 21:1201–1205.

5. Fenn S, Dubern JF, Cigana C, De Simone M, Lazenby J, Juhas M, Schwager S, Bianconi I, Döring G, Elmsley J, Eberl L, Williams P, Bragonzi A, Cámara M. 2021. NirA is an alternative nitrite reductase from *Pseudomonas aeruginosa* with potential as an antivirulence target. MBio 12:2020.12.17.423290.
